# A T3 and T7 Recombinant Phage Acquires Efficient Adsorption and a Broader Host Range

**DOI:** 10.1371/journal.pone.0030954

**Published:** 2012-02-09

**Authors:** Tiao-Yin Lin, Yi-Haw Lo, Pin-Wei Tseng, Shun-Fu Chang, Yann-Tsyr Lin, Ton-Seng Chen

**Affiliations:** Department of Biological Science and Technology, National Chiao Tung University, Hsinchu, Taiwan, People's Republic of China; University of Massachusetts Medical School, United States of America

## Abstract

It is usually thought that bacteriophage T7 is female specific, while phage T3 can propagate on male and female *Escherichia coli*. We found that the growth patterns of phages T7M and T3 do not match the above characteristics, instead showing strain dependent male exclusion. Furthermore, a T3/7 hybrid phage exhibits a broader host range relative to that of T3, T7, as well as T7M, and is able to overcome the male exclusion. The T7M sequence closely resembles that of T3. T3/7 is essentially T3 based, but a DNA fragment containing part of the tail fiber gene 17 is replaced by the T7 sequence. T3 displays inferior adsorption to strains tested herein compared to T7. The T3 and T7 recombinant phage carries altered tail fibers and acquires better adsorption efficiency than T3. How phages T3 and T7 recombine was previously unclear. This study is the first to show that recombination can occur accurately within only 8 base-pair homology, where four-way junction structures are identified. Genomic recombination models based on endonuclease I cleavages at equivalent and nonequivalent sites followed by strand annealing are proposed. Retention of pseudo-palindromes can increase recombination frequency for reviving under stress.

## Introduction

Bacteriophages of the T7 group consist of a large number of related phages that grow on *Escherichia coli* and other bacterial genera. Many coliphages of T7 family have been isolated. Based the on the promoter specificity of phage RNA polymerase and the efficiency of recombination between the phages, T7 group phages that grow on *E. coli* B are classified into three sub-groups [1]. T7 is the prototype of the largest subgroup, the T7-like phages. T3 is the representative of the second subgroup. The third subgroup, the BA phages, contain phages BA 14, BA 127, and BA 156 [1].

The sequence of the genome of T7 (Studier strain) was published in 1983 [2], and that of T3 (Luria strain,T3L) was completed in 2002 [3]. T7 has a linear genome of 39937 base-pairs (bp), while T3 has 38208 bp in the complete genome. It was suggested that they are genetically isolated and not naturally interbreeding populations [4]. Their base sequences display homology across the genomes. Complementation between some phage genes, such as genes *9*, *10*, and *11*, are possible [5]. However, T3 has a number of properties that are not found in T7. T3 is able to escape F plasmid-mediated exclusion, and grow on *E. coli* male strain and *Shigella sonnei* D_2_371-48, while T7 fails to do so [6]. Gene product (gp) 0.3 of T3 possesses an S-adenosyl-methionine hydrolase (SAMase) activity not present in the T7 gp 0.3 [7]. Despite the fact that RNA polymerases from T3 and T7 resemble each other, they can not efficiently transcribe the heterologous DNA [8]. T3 and T7 recombine at a low rate, probably due to mutual exclusion [9], although hybrid T3/7 phages have been isolated [10], [11], [12]. The mechanism of recombination between two T7 group's phages remains unclear. Repair of the T7 double strand breakage (DSB) using a 2.1 kb PCR fragment as a donor resulted in the incorporation of a patch of the donor in vitro, suggesting a strand annealing model of repair [13]. However, a short fragment is especially susceptible to degradation and unwinding, while how a requisite repair patch will be produced for the full-length genome donor is not known. The repair does not require genes 3 (endonuclease I) and 4 (helicase) [13], [14], whereas earlier studies on genetic crosses of mutations in phage T7 have implicated the involvement of genes 2.5 (ssDNA binding protein), 3, 4, 5 (DNA polymerase), and 6 (exonuclease) in T7 genetic recombination [15], [16], [17]. It is a concern that in vitro experiments may overestimate the importance of the patch incorporation pathway [13], [14].

Two different T7 with different phage buoyant density have been reported, T7 Meselson (T7M) and T7 Luria (T7) [4], [18]. Davison and Freifelder also found that these two strains differ in electrophoretic mobility [18]. Both phages were found to have equal DNA length [4]. Davis and Hyman constructed the heteroduplex of T7M (obtained from the Caltech stock) and T3L. Extensive partial sequence homology was observed by electron microscopy. The degree of formation of heteroduplex decreased with increasing formamide concentration. The sequence of T7M has not been reported.

Phages rely on specific hosts for propagation. Unraveling the host range is vital to understanding the interplay between host and virus in the microbial community and its evolutionary consequence. In this study, we show that the propagation of a T7M phage varies on different host strains of *E. coli*, not conforming to the features of T7. Restriction mapping and partial sequencing reveal the close homology of T7M to T3. A T3 and T7 hybrid phage, T3/7, displays yet a different growth pattern. T3 adsorbed inefficiently to a refractory host strain. T7, despite efficient adsorption, was excluded by several host strains. The T3/7 recombinant phage gains efficient adsorption and also a broader host range. Crucial elements rendering the crossover within a small region of homology between T3 and T7 phages were identified, and the recombination model was proposed.

## Results and Discussion

### Propagation on female strains

Plating efficiency of T7, T3, T7M and T3/7 on female strains of *E. coli* was studied. All four phages can grow efficiently on BL21 to a titer of approximately 1×10^10^ ml^−1^ or above. Their plaque sizes are similar (∼0.4 cm). On infecting another female strain DH5α, the efficiency of plating (EOP) is 0.57 for T3, 0.67 for T7M, and about three-fold lower for T3/7 than that of T7M (Table 1). In contrast, T7 has an EOP lower than 10^−6^. The sizes of plaques of T3 and T7M are similar for the phage growing on BL21 and DH5α (∼0.4 cm), but the size (∼0.2 cm) of T3/7 on DH5α is only half of that on BL21.

**Table 1:** Efficiency of plating of phages on *E. coli* female and male strains determined at 37°C.

	EOP			
	T3	T7	T7M	T3/7
BL21	1	1	1	1
K91	<10^−6^	(8±6)×10^−3^	(2.7±2.4)×10^−3^	(8.3±1.7)×10^−1^
DH5α	(5.7±0.7)×10^−1^	<10^−6^	(6.7±2.3)×10^−1^	(2.3±1.0)×10^−1^
XL1-Blue	(5.1±0.5)×10^−1^	<10^−6^	(8.7±1.8)×10^−1^	(6.4±2.5)×10^−1^

### Propagation on male strains

The ability of phages to infect the male K91 strain was tested. T3, T7, and T7M all displayed poor plating efficiency on K91. The EOP of T3 was less than 10^−6^. T7 and T7M displayed very small plaques on K91, ∼0.1 cm or smaller in diameter compared to ∼0.4 cm when propagated on BL21, with EOP approximately around 10^−3^ relative to BL21 (Table 1). Although plaques are quite clear at high phage concentrations (10^7^–10^9^ phages/ml), the numbers of the plaques are not so consistent at phage concentrations below this range. In contrast, T3/7 propagated on K91 with approximately the same EOP and plaque size as on BL21 (Table 1).

The plating efficiency of phages on another male strain, XL1-Blue, was also tested. T3 shows an EOP of 0.51, but EOP of T7 is smaller than 10^−6^. T7M has a high EOP of 0.87 on this strain, albeit the plaques (∼0.15 cm) are smaller than on BL21 (Table 1). EOP of T3/7 on XL1-blue is 0.64, and the plaque sizes (∼0.3 cm) are closer to those on BL21.

### Mapping of phage DNA

**Figure 1:**
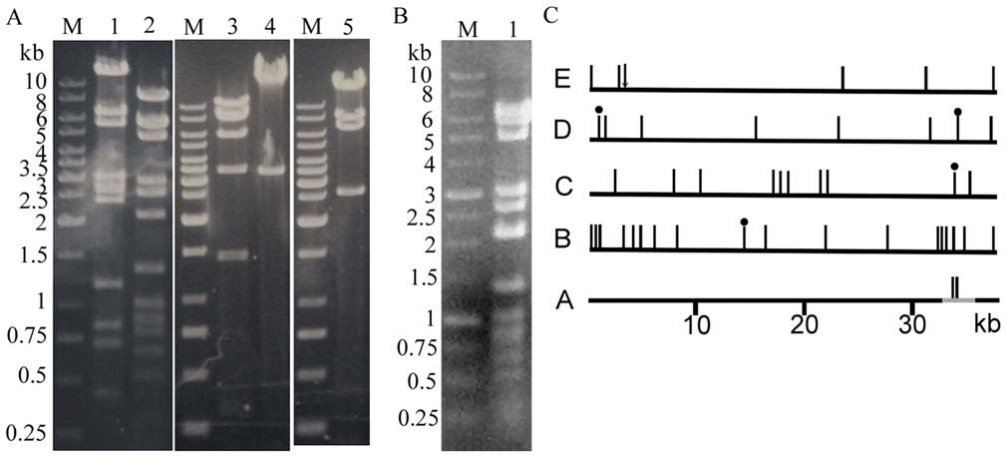
Mapping of the T3/7 and T7M DNA. The genomic DNA was digested by restriction endonucleases, and the fragments were observed by 1% (T3/7) or 0.8% (T7M) agarose gel electrophoresis. M: DNA marker. (A) Digestions of T3/7 DNA. Lane 1, *Mbo*I; lane 2, *Hpa*I; lane 3, *Nde*I; lane 4, *Stu*I; lane 5, *Avr*II. The markers are the same for the three slices of gels. (B) Digestion of T7M DNA. Lane 1, *Hpa*I. (C) Restriction sites in the T3/7 genome. Restriction sites are represented by vertical bars above the DNA. A: The T3 genome (black) and the region replaced by T7 DNA (in grey) in T3/7. The region of recombination is deduced from restriction mapping and sequencing. The two *Mbo*I sites in T7 that replace the single *Mbo*I site at T3 nt 34033 are shown by vertical bars. B to E show restriction sites in T3 genome (black); a dot above the bar indicates that the cutting site is present in T3 but not in T3/7. B: *Hpa*I sites, C: *Mbo*I sites, D: *Nde*I sites, E: *Avr*II cutting positions are indicated by bars, and the single *Stu*I site is shown by an arrow.

Restriction mapping of T7M and T3/7 was used to gain insight into the complicated growth phenomena. T3/7 DNA was digested by *Avr*II, *Hpa*I, *Mbo*I, *Nde*I, and *Stu*I, and the fragments were separated by electrophoresis (Figure 1A). Comparison to the sizes of restriction fragments generated by these enzymes on T3 and T7 (Table S3) shows that the restriction pattern of T3/7 genome was closer to that of T3 than T7, suggesting that the major part of the genome is from T3. Alterations of several nucleotide positions (indicated by nt below) in the T3/7 genome relative to T3 were observed (Figure 1C). *Mbo*I digestion of T3/7 DNA missed the 1423 bp fragment, but showed extra ∼400 bp and ∼1100 bp bands, indicating changes around nt 34033 to nt 35456 that create an extra cutting site. *Nde*I digest of T3/7 DNA missed the 698 bp, 718 bp, 2390 bp, and 3574 bp bands, but at ∼1400 bp and ∼6000 bp two extra bands appeared, indicating base changes near nt 718 and nt 34330 which abolished the two restriction sites. Bands produced by *Avr*II and *Stu*I digestions were the same as predicted for T3.

The *Hpa*I digestion was shown to provide positive identification of T7 and related phages [6]. *Hpa*I fragments of the T3/7 DNA resemble those of T3 except an extra ∼8300 bp band and loss of 2098 bp and 6191 bp bands (Figure 1A, lane 2), indicating that the cutting site at nt 14303 was eliminated in T3/7 relative to T3 (Figure 1C). Unexpectedly, *Hpa*I digestion of the genomic DNA from T7M gave bands distinctly different from those predicted from T7 genome, but consistent with those of T3 (Figure 1B, Table S3).

### Partial nucleotide sequences of T7M phage DNA

The high similarity of T7M to T3 was further confirmed by partial sequencing of T7M DNA. Twenty two percent of the T7M genome was sequenced. The sequenced regions of T7M DNA span 10 different areas on the genome of T7M (Table S4). All the sequences are more homologous to T3 than T7. Compared to the published T3 sequence, only two base substitutions at gene *10B* were found (Table S4). One was a T to C change at nt 22151, which caused an Thr for Ile substitution at residue 421 of gene *10B*. The other was an alteration of T to C at nt 22169, which retains the same amino acid.

### Partial nucleotide sequences of T3/7 phage DNA

Approximately 25% of the T3/7 genome was sequenced. It covers 16 different areas of the T3/7 DNA, encompassing the regions of possible changes as revealed by the restriction mapping and other randomly chosen regions (Table S5). The results demonstrate that the T3/7 DNA is largely based on T3 sequence except that a fragment is exchanged to that of the T7 sequence. However, several regions outside of the exchanged segment showed sporadic nucleotide substitutions compared to the published T3 sequence. These were found in front of gene *0.3*, and in gene *1.1*, gene *2.5*, gene *5*, gene *6*, as well as gene *10B*, and in the region between gene *10B* and gene *11*. The important parts of the sequence are described below.

### The left arm of T3/7 is derived from T3

The sequences of T3/7 nt 26 to 457, and nt 492 to 1008 reveal that the terminal repeat, the *coli* RNA polymerase promoters A0, −35 signal of A1, A2, A3, and the phage promoter φOL as well as the RNaseIII recognition site R*0.3* are all identical to those of T3. In this region only two nucleotides, located between A1 and A2, and between A3 and R*0.3*, respectively, differ from those of the T3 phage, indicating that the left arm of the phage genome is derived from that of T3. The base substitution between A3 and R*0.3* results in the absence of an *Nde*I site at nt 718 of T3/7. One salient difference between T3 and T7 is the presence of SAMase encoded by gene *0.3* (*sam*, nt 901–1359 of T3) in T3, but not in T7 [7]. Partial sequence of the gene *0.3* of T3/7 indicates that *sam* is present in the T3/7.

### Gene *1.2* for escaping F exclusion is present in T7M and T3/7

The presence of the F plasmid *pif*A protein elicits the F exclusion when T7 infects *E. coli* [19], [20]. The T7 gp1.2 or gp10 is necessary for the exclusion, while T3 gp1.2 can prevent the F exclusion [20]. Between T3 and T7, the N terminus of gene *1.2* retains homology, while the distal half exhibits low sequence homology. The complete nucleotide sequences of gene *1.2* of the T7M and T3/7 phages are identical to that of the T3. Therefore, the male exclusion of T7M and the growth difference between T7M and T3/7 are not caused by gene *1.2*.

### Amino acid substitutions occur in gp5 DNA polymerase of T3/7

Phage T7 gp5 is a DNA polymerase with polymerase, exonuclease, and thioredoxin binding domains (TBD) [21]. The host protein thioredoxin (Trx) binds to gp5 and stimulates its polymerase and 3′ to 5′ exonuclease activity [12]. The complete nucleotide sequence of T7M gene *5* is identical to that of T3 gene *5*. T3/7 phage gene *5* incurs three base substitutions (nt 13784, 13956, 14303) and residue changes, A248T, A305V, and N421D, compared with that of T7M. The mutation at nt 14303 results in the loss of a *Hpa*I site at this position (Figure 1). Residues 248 and 421 are located in the polymerase domain, and residue 305 is on the TBD, although not in direct contact with Trx, as illustrated by the crystal structure [21]. Whether the substitutions impose any effect on the gp5 awaits further studies. However, the efficient growth of T3/7 on BL21 suggests that the substitutions do not significantly affect propagation of the phage.

### An amino acid change in the capsid protein from T3 to T7M and T3/7

Genes *10A* and *10B* encode viral capsid proteins. Partial sequences of gene *10A* and *10B* of T7M and T3/7 show high similarity to the counterpart sequences of T3 except for a couple of variations (Tables S4, S5). Most of these changes do not alter the amino acids. However, the T to C substitutions at nt 22151 in both T7M and T3/7 changed the residue 421 of gp10B from Ile to Thr. A –1 frameshift during translation near the end of the coding sequence for the major capsid protein gp10A produces the minor form gp10B in wild-type T7 and T3 phages [2], [22]. T3 gp10A contains 347 residues. Gp10B is 86 residues longer than gp10A. Phage particles normally contain both gp10A and gp10B, but gp10B is dispensable for phage growth [22]. Both T3/7 and T7M could propagate efficiently on BL21, indicating that the substitution of Thr for Ile421 in gp10B does not impair the phage growth. Nevertheless, the possibility of the substitution affecting the structure of the protein or the capsid awaits detail structural investigation.

### Difference in the stem-loop structures of Tφ between phages

Compared to T3L, a G to A replacement at nt 22374 of the T3/7 DNA is located between gene *10B* and gene *11*, while T7M retains a G at this position. After the coding sequence of gene *10B*, a transcription termination signal, Tφ, for T3 RNA polymerase is located between nt 22352–22390. The RNA transcript can form a stem-loop structure at this terminator (Figure S1A). No base changes were observed when sequenced up to nt 22385 for the T7M terminator, and the T7M terminator is expected to have the same structure as that of the T3, which comprises a 17 bp stem and a 5-nucleotide loop. Substitution of A for G at nt 22374 in the T3/7 terminator results in one less base pairing at the top of the stem, and 2 more nucleotides in the loop (Figure S1B). Tφ in T7 has a 15 bp stem, and a 6-nucleotide loop (Figure S1C) and is inefficient in terminating transcription by T7 RNA polymerase [2]. Whether the stem-loop structural difference between these phage terminators has an effect on the termination efficiency requires further investigation.

### A large fragment of DNA changed to the T7 sequence in phage T3/7

**Figure 2:**
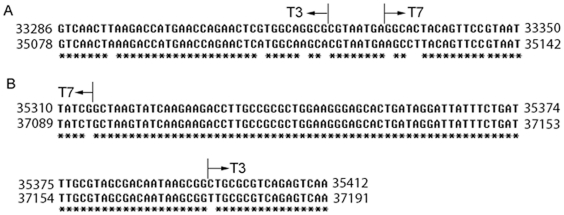
Alignment of T3 and T7 sequences near the crossover regions seen in the T3/7 phage. (A) Within gene *17*. (B) Within gene *18.5*. Sequences were aligned by ClustalW. The parent phages of the two sides of the crossover region (shown by arrows) are indicated on top of the alignment.

A large piece of sequence different from the T3 DNA was identified in T3/7. The sequencing results indicate that the sequences of T3/7 nt 33146–33331 and nt 35382–35936 are indistinguishable from those of T3. However, between these two regions (T3/7 nt 33332–35382), except for a fragment of identical sequence between T3 and T7 (T3 nt 35315–35395 is identical to T7 nt 37094–37174), T3/7 changes to the T7 sequence. This is demonstrated by the identity of T3/7 nt 33332–33638 and nt 34788–35329 to T7 nt 35124–35430 and nt 36580–37121, respectively (Table S5), as well as the consistence of the mapping to T7 sequence rather than T3 sequence in this region. Two smaller *Mbo*I fragments replace the 1423 bp one, as the DNA exchange gives *Mbo*I sites at nt 33893 and nt 34296, in agreement with the T7 sequence, while eliminating the T3 *Mbo*I site at nt 34033 (Figure 1A & C, Table S3). Moreover, T3 DNA has a *Nde*I site at nt 34330, yet the exchanged T7 fragment does not possess a equivalent site, resulting in a ∼6 kb fragment instead of two smaller ones (Figure 1A & C, Table S3). The retention of the *Mbo*I site at T3 nt 35456, and the *Hpa*I sites at T3 nt 34116 and 35125 that have equivalent restriction sites in the T7 DNA, are all consistent with the sites of exchange (Figure 1, Figure 2).

The T3/7 DNA sequence switches from T3 to T7 at nt 33332, and reverts from T7 to T3 sequence anywhere between nt 35302–35382, where the sequence is identical to both T3 and T7 [2], [3]. The exchanged genes are part of gene *17*, complete genes *17.5* and *18*, and part of gene *18.5* and gene *18.7*.

### The recombinant tail-fiber protein of T3/7

Tail fiber proteins participate in the adsorption of phages to the bacterial outer membrane. Bacterial lipopolysaccharide (LPS) was shown to be the receptor for phage T3 and T7 [23]. T3 and T7 tail-fiber proteins encoded by gene *17* consist of 558 and 553 residues, respectively. Each of the six tail-fibers is comprised of a trimer of gp17 [24]. The amino acid sequences of the N-terminal domain (residues 1–149) that interacts with the tail-tube have high homology between T3 and T7, only residues 48 and 118 are different, both incur an Ala to Thr substitution from T3 to T7. Whether the Ala and Thr exchanges have any effect on the interaction with the tail-tube is not clear. Nevertheless, T3/7 has Ala at positions 48 and 118, ensuring proper connection with the tail-tube of the T3 type in the hybrid phage. Sequences of gp17 beyond residue 149 contain antigenicity and the host range determinants, and display less homology (77% identity) between T3 and T7 [24]. Residues 150 to 167 are identical between gp17 of T3 and T7. T7 sequence in T3/7 commences from nt 33332, which resides in the codon for residue 167. Therefore, the antigenecity of T3/7 is expected to be similar to phage T7. The host range of the T3/7, however, is different from T7. The T3/7 hybrid can efficiently infect male *E. coli* including K91 (Table 1), showing that the hybridization has broadened the host range compared to T3 and T7.

### Efficiency of phage adsorption to bacteria

Adsorption of T3/7 to bacteria was compared to the T3 and T7 to investigate the effects of the alteration of tail fiber. Despite the low EOP of T7 on K91, DH5α and XL1-Blue, the efficiency of adsorption of T7 on these strains is higher than 95%, indicating the full competence of the T7 tail fiber in the receptor adsorption (Table 2). The low EOP of T7 on K91 and XL1-Blue can be explained by F exclusion. However, the cause of the low propagation efficiency of T7 on DH5α remains to be investigated. A high adsorption efficiency of 98% but low EOP was also observed for T7M infecting K91, suggesting other factors are affecting the T7M propagation. The efficiency of adsorption of T3 is much worse than T7. The adsorption efficiency of T3 is 46% on XL1-Blue, and plunges to 29% on K91 and DH5α. The deficiency of adsorption of T3 to K91 may contribute to a portion of the low EOP. As T3 has the same low adsorption efficiency on DH5α, yet still exhibits an EOP of 0.51, other undiscovered components may also suppress the propagation of T3 on K91. Compared to T3, the recombinant T3/7 gains a substantial increment in adsorption to K91, DH5α, and XL1-Blue. The efficiency of adsorption to K91 rises from 29% for T3 to 88% for T3/7. The increase in EOP of T3/7 compared to T3 on K91 is more than five orders, among which the proportion conferred by the adsorption increment awaits further elucidation.

**Table 2:** Adsorption efficiency (%) of phages on *E. coli* female and male strains.

	Adsorption efficiency (%)			
	T3	T7	T7M	T3/7
BL21	75±4	86±5	95±2	78±5
K91	29±9	96±4	98±1	88±2
DH5α	29±5	95±1	94±1	85±4
XL1-Blue	46±4	97±1	95±1	84±5

### T7 gene *17.5* and gene *18* can substitute for those of T3 in the T3/7 genome

The gene *17.5* encodes a lysis protein, holin, of 67 residues [2], [3]. Between T3 and T7, the nucleotide sequences of gene *17.5* have 87% identity. Only two amino acids are different between the T3 and T7 gp17.5. Substitution of T7 gp17.5 for T3 gp17.5 in T3/7 does not affect the lysis function since T3/7 lyses BL21 normally.

Gene *18* encodes a non-capsid DNA packaging protein A (terminase small subunit) [2], [3]. Gp18 exhibits 93% identity between T3 and T7, with the carboxyl terminal residues 34–89 completely identical. The gp18 and gp19 complex, which has a prohead-stimulated, DNA-dependent ATPase activity, is required for DNA packaging [25]. The ability of T3/7 to grow efficiently in BL21 indicates that phage DNA packaging proceeds normally. The replacement of gene *18* in T3/7 does not impede the DNA packaging, showing that gp18 from T7 can complex with gp19 from T3 in packaging DNA in vivo. It was also reported that gp18 from T3 or T7 was able to complement the gene *18* ^−^ extract in packaging DNA of either phage into the prohead in vitro [25].

### N-termini of T3 gp18.5 and gp18.7 can be substituted by T7 counterparts in the T3/7 genome

Both genes *18.5* and *18.7* are involved cell lysis. Gene *18.5* encodes a protein of 143 residues for T7 and 147 residues for T3, with 90.5% identity. The protein is an analog of phage lambda protein Rz, which might be an inner membrane murein-specific endopeptidase [26]. Gene *18.7* overlaps with gene *18.5*, but starts from +1 frame. Gene *18.7* encodes an 83-residue protein analogous to lambda protein Rz1, an outer membrane lipoprotein involved in lysis [27]. The C-terminal 10 residues of gp18.7 interact with the C-terminal 50 residues of gp18.5 in T7 [28]. The gp18.5–gp18.7 complex was proposed to lead to fusion of the inner and outer membrane and the final process of bacterial lysis [29]. The T3/7 phage reverts to T3 sequence between nt 35315–35395, which is before the sequences of interaction domains for gp18.5 and gp18.7, thereby securing the proper protein complex formation and the subsequent lysis event. Despite the T3 to T7 DNA replacement causing T48A, A49K, N56D, E57A, and I58V substitutions in gp18.5, and two residues alterations, K19R and R21L, in gp 18.7, T3/7 formed normal plaques in the BL21, indicating that 86 N-terminal residues of T3 gp18.5 and 47 N-terminal residues of T3 gp18.7 can be substituted by their T7 counterparts without hampering the propagation of T3/7.

### T3 gp7.3 is functional with the heterologous T3/7 tail fiber protein

The phage gene *17* tail fiber protein is the main determinant of adsorption and specificity toward bacteria. However, gene *7.3* is also required for T7 to form plaques on *E. coli* B and K12 strains [30]. Gp7.3 is a host specificity protein located on the tail. Without gp7.3 the virion can assemble, yet the assembled tail fiber does not adsorb to the bacteria [30]. The protein may assemble the tail and tail fiber in a proper state for adsorption.

Differences between gp7.3 of T7 and T3 were observed. Gp7.3 is 99 residues in T7 phage and 106 residues in T3 phage. Their amino acid sequences display significant variations with about 56% identity. Whether a heterologous gp7.3 and gp17 combination is functional is an interesting question. The sequencing data demonstrate that both T7M and T3/7 harbor the T3 gp7.3. The ability of T3/7 to infect B and K12 strains efficiently demonstrates that the T3 gp7.3 can function together with the T3/7 hybrid tail fiber proteins for adsorption and infection.

### Crossover sites and advantages gained by the T3/7

T3/7 nt 33332 is the third base of the codon for Glu167 in gp17. Its substitution from G to A (nt 35124 of T7) starts the T7 sequence in T3/7. The sequence of nt 33324 to 33331 are identical between T3 and T7, and bases immediately in front of it vary between these two phages (Figure 2A). Therefore, the crossover in gene *17* happened within the short stretch of 8 nucleotides. The crossover back to the T3 sequence occurred in the nucleotides encoding gp18.5 residue 60 to 86 (T3 nt 35315–35395, equivalent to T7 nt 37094–37174, Figure 2B).

The crossover sites reveal several advantages gained by the T3/7. It was shown that a T3/7 hybrid phage with heterologous terminal redundant regions can not be replicated effectively [31]. T3 and T7 package homologous DNA more efficiently than heterologous DNA. Gp19 is involved in the specificity for packaging the homologous DNA, and the DNA sequence responsible for recognition is located within 5% of the termini of the phage genomes [32]. Thus in the present T3/7 phage, the crossover in gene *17* can enhance adsorption to certain hosts, and that in gene *18.5* can assure that gene *19* and the right end terminal DNA retain the sequences of the T3 phage.

During transcription, phage RNA polymerase interacts with two phage proteins, gp3.5 (lysozyme) and gp19 [33]. The polymerase-gp3.5 complex pauses at the right end of the concatemer junction and interacts with gp18 and gp19 to initiate maturation and DNA packaging [34]. By returning back to the T3 sequence within gene *18.5*, T3/7 can retain the efficient interaction between the RNA polymerase and gp19.

Gp19 also binds to the prohead of phage. Residues 571 to 576 of gp19 form the core domain crucial for binding the prohead, and the C-terminal ten residues, 577 to 586, form the anchor domain for stable binding [35]. These amino acids display low identity between T3 and T7. Thus, the change back to gene *19* of T3 can also benefit T3/7 by allowing a stable complex between gp19 and the prohead.

In light of the above multiple functions of gp19, the reversion to T3 gene *19* sequence may be a prerequisite for the packaging and maturation of the hybrid phage.

### Identification of four-way junction structures for Endo I cleavage

**Figure 3:**
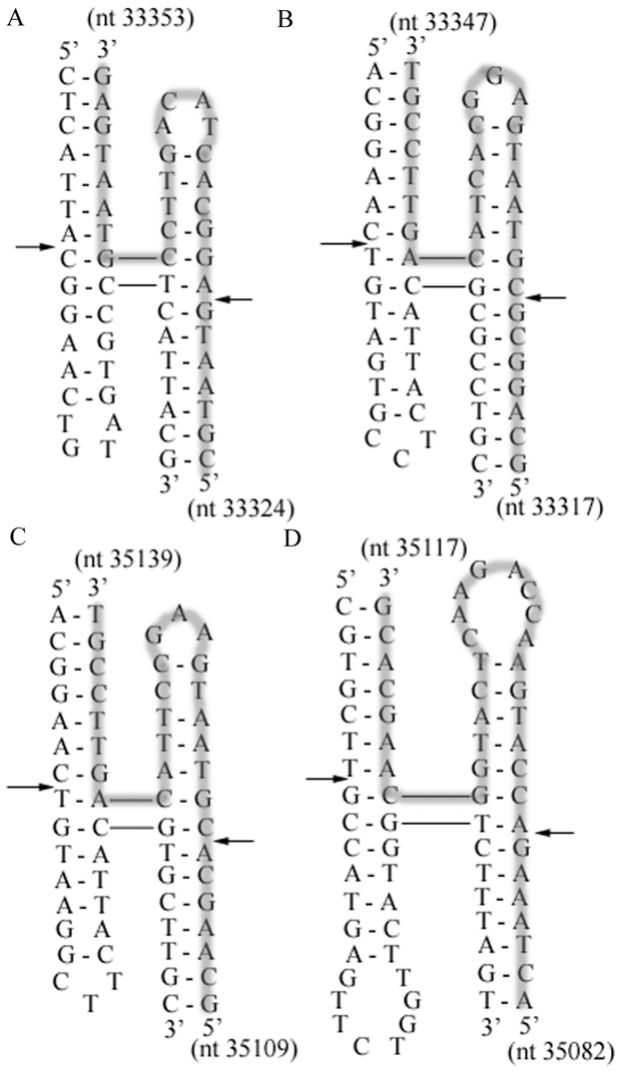
Structures of four-way junctions in gene *17* of T3 and T7. (A) T3 nt 33324–33353 (B) T3 nt 33317–33347 (C) T7 nt 35109–35139 (D) T7 nt 35082–35117. One strand is highlighted in grey; the other is not. Arrows indicate Endo I cutting sites.

To understand the mechanism of recombination within the short 8 nucleotides, we identified that the nearby nucleotides can form four-way junction structures [36] (Figure 3). Such structures are apt to be cut by phage endonuclease I (Endo I) [36]; for instance in Figure 3A, the cutting sites are primarily between nt 33330 and 33331 for the sense strand and between nt 33346 and 33347 for the antisense strand.

**Figure 4:**
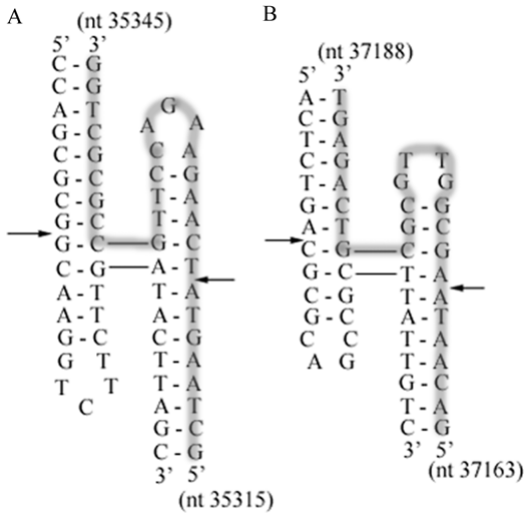
Structures of four-way junctions in gene *18.5* of T3 and T7. (A) T3 nt 35315–35345 or equivalently T7 nt 37094–37124 (B) T7 nt 37163–37188. One of the strands is highlighted in grey. Endo I cutting sites are shown by arrows.

The other crossover occurs within gene *18.5*. Search for four-way junctions for Endo I cleavages identified prominent areas around T3 nt 35315–35345 (sequence identical to T7 nt 37094–37124) and T7 nt 37163–37188 (Figure 4). Endo I does not exhibit a clear minimal arm length requirement at a junction; a duplex arm of 4 base pairs or shorter was cleaved [36], [37]. The cleavage is essentially structure specific and prefers non-crossover strands at branched junctions [37].

### Recombination models

The previous in vitro experiment shows efficient patch incorporation of a donor fragment in T7 DSB repair [13]. It suggests that a T7 fragment, which covers the exchanged area, can serve as a donor for T3 DSBs to yield the recombinant T3/7.

**Figure 5:**
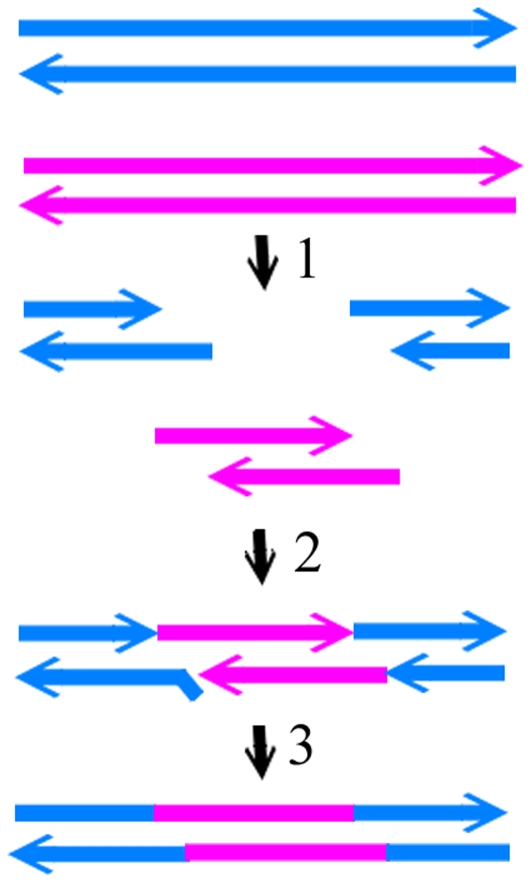
The mechanism of endonucleolytic cleavages at equivalent sites and strand annealing (CESA). T7 and T3 DNA are shown in blue and pink, respectively. Arrows indicate the 3′ end. The steps are: 1, cleavage of T3 and T7 genomes by Endo I at equivalent sites; 2, annealing of the DSBs at both ends of T3 genome with the T7 DSB from the middle of the genome; 3, displacement and removal of the nonhomologous region, then filling the gap and ligation of the ends.

Separately, the short stretch of homology and special sequence in the crossover site also shed light on the recombination path. The identification of the four-way junctions in this region suggests a role of the Endo I cleavage in phage recombination. Seeing that the ends generated by Endo I cleavages of the 8 bp homology regions in both T3 and T7 can produce complementary pairing, a simplest model is proposed based on cleavages at equivalent-sites of phage DNA followed by strand annealing (CESA) (Figure 5). The available sequences allow a closer examination of whether this and the following (see below) proposed mechanisms will produce sequences at the crossover sites matching those of the recombinant T3/7. Endo I cleavages at four-way junctions produce DSBs. After cutting the structures in the equivalent positions of T3 (Figure 3B) and T7 (Figure 3C), the 5′ protruding tail of T7 DSB will comprise of 8 bases that complement T3 DSB nt 33324–33331, followed by 9 bases in incomplete homology with T3 5′ overhang nt 33332–33340 (Figure 5). Phage gp2.5 is a recombinase that can mediate single strand annealing [38]. The incomplete homologous T3 5′ overhang is displaced and cleaved by T7 (or T3) gp6 exonuclease [39]. The gap is refilled by T7 gp5/Trx or *E. coli* DNA polymerase I using the T7 tail as a template, and then the ends are ligated [40], [41].

The Endo I cutting sites (Figure 4A) in gene *18.5* are within a longer homologous region (Figure 2), and therefore the 5′ protrusions of the DSBs generated from T7 and T3 genomes can be directly annealed through 15-base complementarity and then ligated (Figure 5). T7 gp2.5 has a high efficiency in stimulating DNA annealing, much higher than T4 gene *32* protein and *E. coli* SSB protein [38]. T3 DNA ligase has an efficiency 5 to 6-fold higher than T4 DNA ligase for cohesive DNA fragments. In vitro ligation of 4.5×10^−8^ M *Hin*dIII cut fragments using 0.1 nM T3 DNA ligase achieved 90% ligation in 30 min without gp2.5 [42]. Phage gp2.5 and ligase thus can promote the recombination reaction through the strand annealing mechanism.

**Figure 6:**
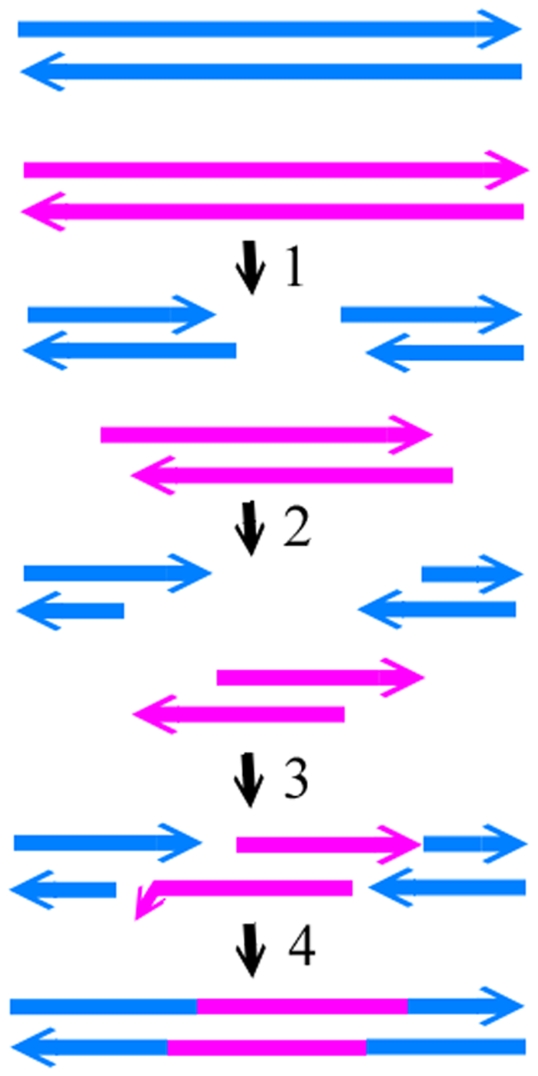
The mechanism of endonucleolytic cleavages at nonequivalent sites and strand annealing (CNSA). Colors and arrows are the same as those of Figure 5. The steps are: 1, production of DSBs by cutting at nonequivalent sites of T3 and T7 DNA; 2, 5′ resections of DSBs of T3 and T7; 3, annealing of the T3 DSBs with the T7 DSB; 4, removal of the nonhomologous nucleotides, filling the gap, and ligation.

Cutting at equivalent positions in two phages is not the only way to produce two DSBs capable of annealing. Alternatively, recombination can also proceed through cleavages at nonequivalent-sites of DNA followed by strand annealing (CNSA) (Figure 6). For instance, two four-way junction structures were identified to be in nonequivalent positions in gene *17* of T7 and T3, where the structure in T7 (Figure 3D, T7 nt 35082–35117) lies upstream of that in T3 (Figure 3A, T3 nt 33324–33353). Endo I cleavages of both structures result in DSBs that can be annealed after 5′ resections (Figure 6). The T3 DSB 3′ tail (nt 33324–33330) can anneal with the T7 DSB 3′ nt 35116–35122 through the 7-base pair complementarities. The 5 nucleotides (T7 nt 35111–35115) at the 3′ end of T7 DSB are not completely homologous with T3 nt 33319–33323, and therefore are excised with the gap refilled. The 3′ to 5′ exonuclease activity and polymerase activity of gp5/Trx or *E. coli* DNA polymerase I can carry out the excision and gap-filling [40], [41]. Similarly, within gene *18.5*, a T7 four-way junction structure (Figure 4B, T7 nt 37163–37188) lies downstream of the position of the T3 four-way junction structure (Figure 4A, T3 nt 35315–35345). Endo I cleavages of both structures and 5′ resections of the DSBs result in a T7 DSB with a 3′ protrusion ending at nt 37169 and a T3 DSB with a 3′ tail ending at nt 35338. The 3′ overhangs of the two DSBs are annealed due to the identical sequences between T7 nt 37117–37169 and T3 nt 35338–35390. The remaining gaps are filled and ligated. The frequency of phage recombination can be increased through the possibility of annealing DSBs generated by different Endo I cutting sites.

Homologous recombination invoking strand transfer, such as double Holliday junction formation [43], remains a possible pathway for the production of T3/7. A very low frequency of recombination is expected by utilization of the 8 bp homology for strand transfer, since strand transfer has a minimal requirement of 23–27 bp homology [44]. Nevertheless, except several mismatches, the 8 bp homology occurs in a context of sequences sharing longer homology that has a possibility in giving rise to strand transfer. However, in this case all the mismatches in front of the 8 bp match will need to be repaired selectively to maintain the sequences of T3 to yield the T3/7. A combination of strand annealing and strand transfer reactions is also possible in recombination. Small regions of microhomology have been suggested to be positions of genetic exchange through homologous recombination, although the detailed mechanism was not investigated [45].

Possibility exists that despite the presence of the four-way junctions in the crossover sites of T3 and T7, cleavages were not performed at these positions. Inside the cells, the left-side and right-side DSBs of the T3 genome can be generated by other endonucleases to include the left and right crossover sites, respectively. Similarly a T7 DSB comprising the exchanged fragment can also be generated through cutting sites different from the Endo I sites. Subsequent exonuclease digestion of these DSBs can produce single-stranded regions that can then be annealed to form a recombinant phage. However, for generation of the T3/7 with DSBs cleaved farther away from the crossover sites, the frequency of a) extensive unwinding and exonucleolytic digestion to reach the crossover areas, b) annealing the left-side DSB of T3 with a T7 DSB at only the 8 bp homology, and removing a long overhang with other sequence homology or c) repairing all mismatched positions selectively to obtain a sequence conformable to that of T3 before the 8 bp homology is likely decreased.

### Pseudo-palindromes and Endo I function in recombination

The in vitro patch incorporation for T7 DSB repair was found to require gp6 exonuclease and gp2.5, but was independent of Endo I, helicase, T7 DNA polymerase, and *E. coli* recombinase (RecA) [14]. The study used DSBs produced by *Xho*I cutting at an engineered site and a synthesized donor fragment with no restriction on the incorporation positions, and therefore the importance of Endo I may not be observed. Nevertheless, the in vitro incorporation of a patch of donor is in agreement with the phage recombination models deduced from the microhomology and four-way junction. When the phage genome serves as a donor, Endo I can generate various phage DNA fragments that then anneal directly or after 5′ resections, as proposed in CESA and CNSA, for repair or recombination. This also alleviates the difficulty of unwinding and degrading the long genome for a requisite patch. Under stressed conditions, phages strive by recombination, which eventually leads to the diversity of phages. Phage genomes were found to avoid palindromes [3], yet as observed in this study, retention of pseudo-palindromes in phage may serve functions in recombination and repair.

### Broadening the host range with some compromise

T7 and T3 manifest low plating efficiency on K91, yet the T3/7 phage raises its EOP to near 1. The acquisition of the T3/7 hybrid phage's ability to infect male K91 indicates that the host range is broadened. Interestingly, the EOP of T3/7 on a female strain DH5α is lower than T3 by 2.5 folds. The size of plague is also smaller than those formed by T3 on DH5α. This illustrates that while the host range is broadened, the efficiency of propagation toward some other strains may be compromised. Sequencing results demonstrate that the T7M phage is closely related to T3L. The deficiency in infecting the male K91 strain does not arise from the absence of T3 gene *1.2* or deterred adsorption. Complete genomic sequencing for T7M and T3/7 is underway to explore the possible explanation.

### Applications of Endo I and pseudo-palindromes in genetic and phage manipulation

Our study suggests that phages may have preserved pseudo-palindromes for recombination of genes to survive harsh conditions. It is feasible to implement the same strategy in cloning or targeting a desired DNA sequence for genetic manipulation of cells and isolated DNA via short pseudo-palindromic sequences and Endo I cleavages. Endo I and pseudo-palindromes can also be employed to generate a novel hybrid phage with altered bacterial host range for phage therapy.

## Materials and Methods

### Materials

Restriction enzymes were purchased from New England Biolabs (Berverly, MA), Promega (Madison, WI) and Fermentas (Hanover, MD). Chemicals were from Sigma Chemical Co. (St. Louis, MO). T7M was obtained from ATCC (11303-B38). *E. coli* strain K91 and phage T3/7 were generously provided by Dr. M. Russel (Rockefeller University) [12], [46]. Dr. S. R. Kushner (University of Georgia) kindly supplied *E. coli* SK3967 [47]. T3 and T7 were generous gifts of Dr. F.W. Studier (Brookhaven National Laboratory) [6]. Genotypes of strains used are listed in Table S1.

### Propagation of phages

The titers of T3/7 and T7M were determined on *E. coli* strain BL21. Propagation of phages follows the procedure described previously [48]. Briefly, *E. coli* was grown in T-medium until an OD_600_ of 0.5, and infected with T3/7 or T7M that had been serially diluted. The infected cells were plated on agar plates, and incubated at 37°C overnight. The plaque numbers were counted. Efficiency of plating (EOP) was determined with respect to BL21.

### Efficiency of Adsorption

The measurements of adsorption of bacteriophage to *E. coli* follow the method of Koike and Iida [49] with some modification. Bacterial culture was grown in T medium until OD_600_ of approximately 0.5–0.6, and mixed with a phage suspension of a titer of about 3×10^9^ ml to yield an MOI of 0.5. After 10 min incubation at room temperature, 0.1 ml of the mixture was diluted with 0.9 ml of saline containing 3.3% (v/v) chloroform, and the number of unadsorbed phage was determined by counting the plaque forming units (PFU). The adsorption efficiency was calculated from [1−(PFU of free phage after adsorption/original PFU in the bacteria-phage mixture)]×100%.

### Purification of phage DNA

*E. coli* strain SK3967 carrying thioredoxin gene on a plasmid, pET/trxA, was grown to OD_600_ of 0.5 to 0.6, infected with a phage, and then cultured until the cells were lysed (∼2.5 h). DNase I was added to a final concentration of 0.2 µg/ml. After incubation at 37°C for 15 min, NaCl was added to 2.5% (w/v). The cultures were centrifuged at 12000× g, 4°C for 15 min, and PEG8000 was dissolved into the supernatant to a final 10% (w/v). The solution was kept at 4°C for 8–12 hr. The phage was spun down by centrifugation at 12000× g, 4°C for 15 min, resuspended in 1.5 ml TE buffer (10 mM Tris, 1 mM EDTA, pH 8.0) with 1 M NaCl, and centrifuged again at 11500× g in room temperature for 10 min. A CsCl gradient was prepared by mixing 62.5% (w/v) CsCl and TE buffer in a ratio of 1∶0, 2∶1, 1∶1, and 1∶2, with a volume of 0.5 ml, 1 ml, 1 ml, and 1 ml, respectively. The supernatant was loaded onto the gradient, and centrifuged at 210000× g, 4°C for 2 hr. The phage was removed and dialyzed in 1 liter of 0.1 M Tris, 0.1 M NaCl, pH 8.0. DNA was extracted once by 90% phenol and twice by chloroform/isopropanol (24∶1), and then dialyzed in TE buffer overnight.

### DNA sequencing

Fragments of phage DNA were amplified by PCR using a mix of Taq and Pfu DNA polymerase (Protech Technology Enterprise, Taiwan) and oligonucleotides upstream and downstream of the region to be sequenced as primers. Sequencing was performed by a Perkin-Elmer 377 DNA autosequencer or an ABI 3730XL autosequencer using an ABI prism Dye Terminator Cycle Sequencing Ready Reaction Kit (PE Applied Biosystems, Forter City, CA). The sequences of oligonucleotides used for sequencing are listed in Table S2.

### Sequence analysis

Sequence alignment was performed using *ClustalW*. Repeated sequences were identified via the EMBOSS PALINDROME software [50] and manual inspection.

### Accession numbers

The sequence data of phages T7M and T3/7 were deposited to GenBank with accession numbers JF906059 and JF906060, respectively. We refer to accession numbers NC_001604.1 and AJ318471.1 for the sequences of T7 and T3, respectively.

## Supporting Information

Figure S1 **Stem-loop structures of phage terminator Tφ in T3, T3/7, and T7.** The drawings show the structures in nt 22352–22390 for T3 (A) and T3/7 (B). From the sequenced T3/7 nucleotides in this region, nt 22352–22381, it can be inferred that the G to A replacement at nt 22374 of T3/7 increases the size of the loop of Tφ while reducing one base pair at the top of the stem. The structure of T7 terminator Tφ is shown in (C) for comparison.
(TIF)

Table S1 **Bacterial strains.**
(DOC)

Table S2 **Sequences of primers.** Primers 1–15 were used to sequence T7M DNA. Primers 5–18 were used to sequence T3/7 DNA. Gene *5* from both phages were cloned to a T vector by primers 19–20, and sequenced by primers 21–25.
(DOC)

Table S3 **Restriction sites and fragment sizes of T3 and T7 DNA.** The positions of restriction sites and sizes of fragments generated by restriction endonucleases on T3 and T7 DNA based on published sequences.
(DOC)

Table S4 **Sequenced positions of phage T7M.** The numbering of nucleotides (2^nd^ column), as well as the genes, promoters (φ), terminators (T), and RNase III sites (R) (3^rd^ column), follows that of phage T3L. If a gene is not completely sequenced, the number of nucleotides sequenced from 5′, 3′ or the middle (mid) of the gene is indicated inside the parenthesis. In all sequenced positions, only nt 22151 and nt 22169 are different from T3. Both incur a T→C change in gene *10B*.
(DOC)

Table S5 **Sequenced positions of phage T3/7 and changes relative to T3L.** The numberings of nucleotides (2^nd^ column), genes, promoters (A, φ), terminators (T), and RNase III sites (R) follow those of phage T3L, except that due to the shorter exchanged T7 region, the numbering of T3/7 in column 2 is reduced by 13 compared to T3. For the regions changed to T7, the nucleotide numbering in the T7 genome are indicated in the fourth column, while the multiple base changes are not listed. In the fifth column, a slash indicates the mutation location between the two identities.
(DOC)
