# Supplementary figures and images for: A T3 and T7 Recombinant Phage Acquires Efficient Adsorption and a Broader Host Range
Source: PLoS One. 2012 Feb 9;7(2):e30954. doi: 10.1371/journal.pone.0030954 (PMC3276506; doi:10.1371/journal.pone.0030954)

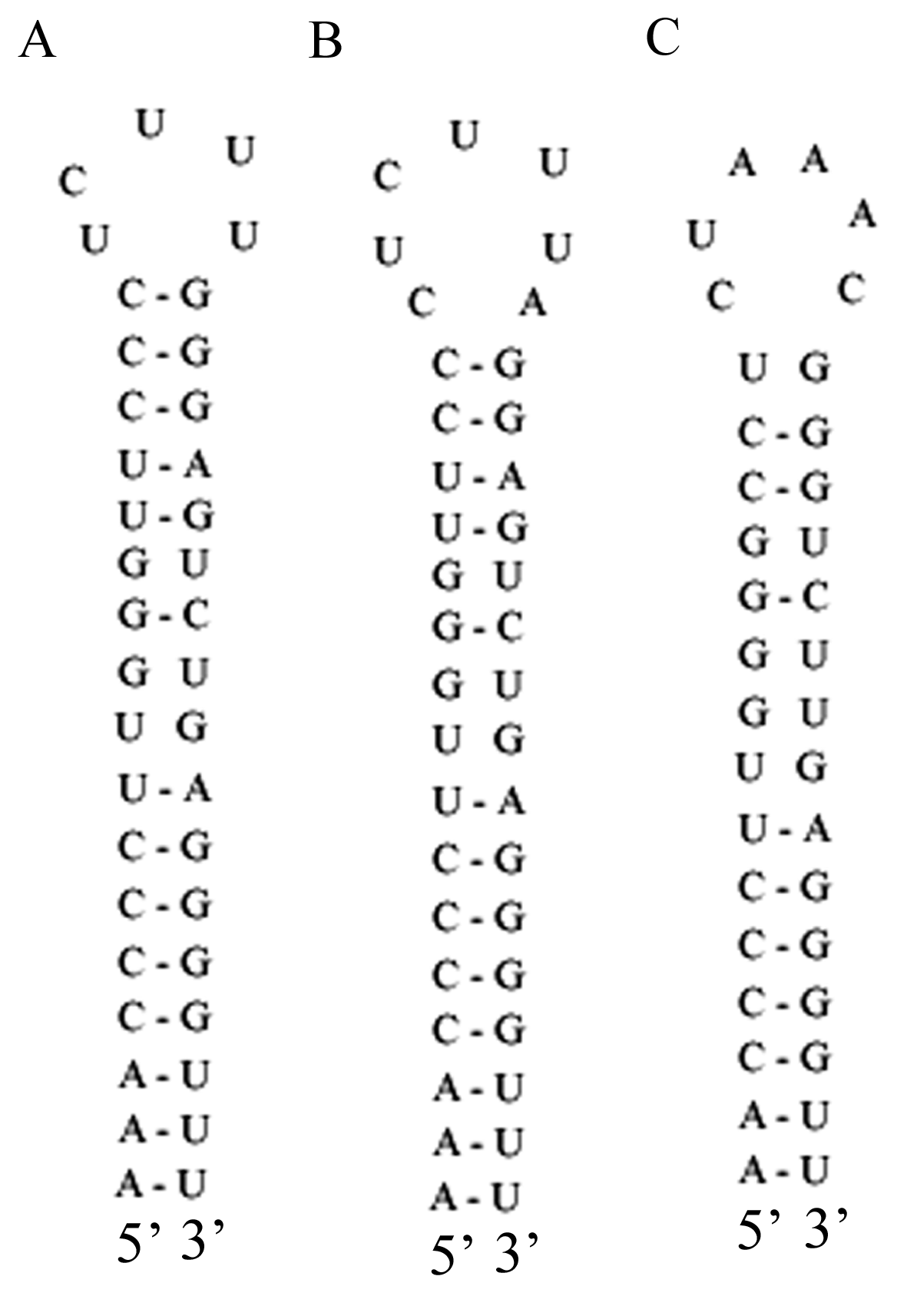

Supplement: Figure S1 — Stem-loop structures of phage terminator Tφ in T3, T3/7, and T7. The drawings show the structures in nt 22352–22390 for T3 (A) and T3/7 (B). From the sequenced T3/7 nucleotides in this region, nt 22352–22381, it can be inferred that the G to A replacement at nt 22374 of T3/7 increases the size of the loop of Tφ while reducing one base pair at the top of the stem. The structure of T7 terminator Tφ is shown in (C) for comparison. (TIF) [file pone.0030954.s001.tif]
