# Supplementary material for: A T3 and T7 Recombinant Phage Acquires Efficient Adsorption and a Broader Host Range
Source: PLoS One. 2012 Feb 9;7(2):e30954. doi: 10.1371/journal.pone.0030954 (PMC3276506; doi:10.1371/journal.pone.0030954)
Supplement: Table S1 — Bacterial strains. (DOC) [file pone.0030954.s002.doc]

| strain | genotype |
| --- | --- |
| BL21 | F^-^ *ompT* *gal* [*dcm*][*lon*]*hsdS*_B_(r_B_^-^m_B_^-^) |
| K91 | HfrC *Sup*^+^ *pho*(Am) |
| DH5α | F^-^ *endA1* *hsdR17*(r_K_^-^m_K_^+^) *glnV44* *thi*-*1* *recA1* *gyrA* *relA1* △(*lacZYA*-*argF*)*U169* *deoR* (φ80 *lacZ*△*M15*) |
| XL1-Blue | F’ *proAB* *lac*I^q^ *Z*△*M15* *recA1* *endA1* *gyr*96 *thi-1* *hsdR17*(r_K_^-^ m_K_^+^) *glnV44* *relA1* *lac* |
| SK3967 | *argE*4, *his*-4, *ilvD*188, *trxA*1 |
