# Supplementary material for: A T3 and T7 Recombinant Phage Acquires Efficient Adsorption and a Broader Host Range
Source: PLoS One. 2012 Feb 9;7(2):e30954. doi: 10.1371/journal.pone.0030954 (PMC3276506; doi:10.1371/journal.pone.0030954)
Supplement: Table S2 — Sequences of primers. Primers 1–15 were used to sequence T7M DNA. Primers 5–18 were used to sequence T3/7 DNA. Gene 5 from both phages were cloned to a T vector by primers 19–20, and sequenced by primers 21–25. (DOC) [file pone.0030954.s003.doc]

| primer | Type or nucleotide position in phage | Sequence |
| --- | --- | --- |
| 1 | 32772-32791 | 5’-TAGGGAGAGGCGAAATAATC-3’ |
| 2 | 34550-34569 | 5’-CATGTGTCCTCCTTAGTGTG-3’ |
| 3 | 12886-12905 | 5’-CTGACTATGGCTGCTGACAA-3’ |
| 4 | 15265-15283 | 5’-TTACGAGAAGACCCTATGC-3’ |
| 5 | 5911-5930 | 5’-CACTGAGGACAACGAAATGA-3’ |
| 6 | 8289-8308 | 5’-ACTTCTGTCTTCGGTGCTTC-3’ |
| 7 | 8813-8832 | 5’-ATCCGTCCAGAAGTGAAAGC-3’ |
| 8 | 10722-10741 | 5’-GTTACCATCAGAAGACCCAC-3’ |
| 9 | 16200-16219 | 5’-CTTGTGATGGATGGAGACTG-3’ |
| 10 | 18139-18158 | 5’-TGTTAGGCGGTCATAGGTAG-3’ |
| 11 | 20925-20944 | 5’-CTAATCAGGGTAAGGGTCAG-3’ |
| 12 | 22405-22424 | 5’-GATGTTACCTCCTGTTAGTG-3’ |
| 13 | 464-483 | 5’-GGCTATTATTCATCTCGTCA-3’ |
| 14 | 464-483 | 5’-TGACGAGATGAATAATAGCC-3’ |
| 15 | 33108-33127 | 5’-ATCCTCCGTGCGTATGACCT-3’ |
| 16 | 35367-35386 | 5’-GTCGCTACGCAAATCAGAAA-3’ |
| 17 | 35367-35386 | 5’-TTTCTGATTTGCGTAGCGAC-3’ |
| 18 | 36993-37012 | 5’-ATCACGAATCACAAGGCGGT-3’ |
| 19 | GV5’EcoRV | 5’-GCTCTAGATATCCAAAGGAGGGCATTATG-3’ |
| 20 | GV3’BamHI | 5’-GCAACGGATCCCATAATACC-3’ |
| 21 | 13943-13962 | 5’-CGCTGTGCCTTGTTCTTAGG-3’ |
| 22 | 13821-13840 | 5’-CGTTCGGCTCGTGGTATCAG-3’ |
| 23 | 14749-14768 | 5’-ACTCGACAAGTGTCTGTTGG-3’ |
| 24 | Forward primer | 5’-CAAGGCGATTAAGTTGGGTA-3’ |
| 25 | Reverse primer | 5’-GGAATTGTGAGCGGATAACA-3’ |
