# Supplementary material for: A T3 and T7 Recombinant Phage Acquires Efficient Adsorption and a Broader Host Range
Source: PLoS One. 2012 Feb 9;7(2):e30954. doi: 10.1371/journal.pone.0030954 (PMC3276506; doi:10.1371/journal.pone.0030954)
Supplement: Table S3 — Restriction sites and fragment sizes of T3 and T7 DNA. The positions of restriction sites and sizes of fragments generated by restriction endonucleases on T3 and T7 DNA based on published sequences. (DOC) [file pone.0030954.s004.doc]

| Restriction enzyme | nucleotide position (nt) on T3 | T3 fragment size (bp) | nucleotide position (nt) on T7 | T7 fragment size (bp) |
| --- | --- | --- | --- | --- |
| *Hpa*I | 92, 320, 617, 3221, 4005, 4656, 5988, 8112, 14303, 16401, 22174, 27799, 32610, 32736, 33243, 34116, 35125, 38069 | 92, 126, 139, 228, 297, 507, 651, 784, 873, 1009, 1332, 2098, 2124, 2604, 2944, 4811, 5625, 5773, 6191 | 2530, 4848, 7309, 7755, 11807, 15613, 17768, 18610, 24519, 24938, 25226, 26966, 29591, 30209, 34528, 35908, 36904, 37816 | 288, 419, 446, 618, 842, 912, 996, 1380, 1740, 2121, 2155, 2318, 2461, 2530, 2625, 3806, 4052, 4319  5909 |
| *Mbo*I | 2380, 7944, 10574, 17018, 17824, 18506, 21500, 22149, 34033, 35456 | 649, 682, 806, 1423, 2380, 2630, 2752, 2994, 5564, 6444, 11884 | 8311, 8414, 11515, 14354, 35684, 36087 | 103, 403, 2839, 3101, 3850, 8311, 21330 |
| *Nde*I | 718, 1416, 4845, 15370, 23340, 31940, 34330, 37904 | 304, 698, 718, 2390, 3429, 3574, 7970, 8600, 10525 | 5676, 6364, 14308, 19956, 22966, 27727, 33732 | 688, 3010, 4761, 5648, 5676, 6005, 6205, 7944 |
| *Avr*II | 72, 2721, 23646, 31431, 38049 | 72, 159, 2649, 6618, 7785, 20925 | 20005, 31564, 33223 | 1659, 6714 11559, 20005 |
| *Stu*I | 3316 | 3316, 34892 | 19022 | 19022, 20915 |
