# Supplementary material for: A T3 and T7 Recombinant Phage Acquires Efficient Adsorption and a Broader Host Range
Source: PLoS One. 2012 Feb 9;7(2):e30954. doi: 10.1371/journal.pone.0030954 (PMC3276506; doi:10.1371/journal.pone.0030954)
Supplement: Table S4 — Sequenced positions of phage T7M. The numbering of nucleotides (2nd column), as well as the genes, promoters (φ), terminators (T), and RNase III sites (R) (3rd column), follows that of phage T3L. If a gene is not completely sequenced, the number of nucleotides sequenced from 5′, 3′ or the middle (mid) of the gene is indicated inside the parenthesis. In all sequenced positions, only nt 22151 and nt 22169 are different from T3. Both incur a T→C change in gene 10B. (DOC) [file pone.0030954.s005.doc]

| Primer | Positions of sequenced nucleotides | Gene, promoter, T, and R sequenced |
| --- | --- | --- |
| 5 | 5950-6963 | *1.05* (3’ 40 nt), φ*1.1*, R*1.1*, *1.1*, *1.2*, φ*1.3*, R*1.3*, *1.3* (5’ 369 nt) |
| 6 | 7614-8267 | *1.3* (3’ 22 nt), T_E_, φ*1.5*, *1.5*, *1.6*, *1.7* (5’ 207 nt) |
| 7 | 8849-9281 | φ*2.5*, *2.5* (5’ 394 nt) |
| 8 | 10263-10667 | *3.5* (3’ 230 nt), *3.7*, φ*3.8*, R*3.8* |
| 3 | 12927-13437 | *4.5* (3’ 49 nt), R*4.7*, *5* (5’ 395nt) |
| 21-23 | 13436-14641 | *5* (mid 1206 nt), *5.1*, *5B* |
| 4 | 14637-15234 | *5* (3’ 78 nt), *5B*, *5.3* (5’ 67 nt) |
| 9 | 16255-16642 | *6* (mid 388 nt) |
| 10 | 17651-18104 | *6.7* (3’ 85 nt), *7.3*, *8* (5’ 12 nt) |
| 11 | 20958-21565 | *10A* (mid 608 nt), *10B* (mid 608 nt) |
| 12 | 21956-22385 | *10B* (3’ 236 nt), Tφ (5’ 34 nt) |
| 1-2 | 32815-34528 | *17* |
