# Supplementary material for: A T3 and T7 Recombinant Phage Acquires Efficient Adsorption and a Broader Host Range
Source: PLoS One. 2012 Feb 9;7(2):e30954. doi: 10.1371/journal.pone.0030954 (PMC3276506; doi:10.1371/journal.pone.0030954)
Supplement: Table S5 — Sequenced positions of phage T3/7 and changes relative to T3L. The numberings of nucleotides (2nd column), genes, promoters (A, φ), terminators (T), and RNase III sites (R) follow those of phage T3L, except that due to the shorter exchanged T7 region, the numbering of T3/7 in column 2 is reduced by 13 compared to T3. For the regions changed to T7, the nucleotide numbering in the T7 genome are indicated in the fourth column, while the multiple base changes are not listed. In the fifth column, a slash indicates the mutation location between the two identities. (DOC) [file pone.0030954.s006.doc]

| Primer | Positions of sequenced nucleotides | Gene, promoter, T, and R sequenced | Position and base mutation relative to T3 | Mutated site |
| --- | --- | --- | --- | --- |
| 14 | 26-457 | Terminal repeat (3’ 206 nt), A0, φ0L, A1 (-35 signal) |  |  |
| 13 | 492-1008 | A2, A3, R*0.3*, *0.3* (5’ 108 nt), *0.3B* (5’ 27 nt) | 569 G→A  718 T→C | A1/A2  A3/R*0.3* |
| 5 | 5958-6521 | *1.05* (3’ 32 nt), φ*1.1*, R*1.1*, *1.1*, *1.2,* φ*1.3* | 6177 C→T | *1.1* |
| 6 | 7708-8255 | *1.5*, *1.6*, *1.7* (5’ 195 nt) |  |  |
| 7 | 8856-9383 | *2.5* (5’ 496 nt) | 8980 G→A | *2.5* |
| 8 | 10168-10714 | *3.5* (3’ 325 nt), *3.7*, *3.8*, R*3.8*, *4A* (5’ 45 nt) |  |  |
| 21-25 | 13028-15183 | *5, 5.1,5B, 5.3 (5’ 16 nt)* | 13784 G→A  13956 C→T  14303 A→G | *5, 5B* |
| 9 | 16247-16817 | *6* (mid 571 nt) | 16436 G→A | *6* |
| 10 | 17567-18082 | *6.7* (3’ 169 nt), *7.3* | 17749 T→C | *6.7*/*7.3* |
| 11 | 20945-21470 | *10A* (mid 526 nt), *10B* (mid 526 nt) |  |  |
| 12 | 21816-22381 | *10A* (3’ 119 nt), *10B* (3’ 376 nt), Tφ (5’ 30 nt) | 22151 T→C  22169 T→C  22299 G→A  22374 G→A | *10B*  *10B*  *10B*/*11*  Tφ |
| 15 | 33146-33638 | *17* (mid 493 nt) | 33332-33638 →T7 nt 35124-35430 | *17* |
| 16 | 34788-35329 | *18* (3’ 243nt), R*18.5*, *18.5* (5’ 205 nt), *18.7* (5’ 90 nt) | 34788-35329  → T7 nt 36580-37121 | *18*, R*18.5*, *18.5*, *18.7* |
| 17 | 35382-35936 | *18.5* (3’ 187 nt), *18.7* (3’ 110 nt), *19* (5’ 359 nt) | 35382-35936→T3 nt 35395-35949 |  |
| 18 | 36603-36968 | *19* (mid 366 nt), *19.2* (3’ 62 nt), *19.3* | 36603-36968→T3 nt 36616-36981 |  |
